# Adenomyoma of the Rectum

**Published:** 1917-12

**Authors:** Frank C. Yeomans

**Affiliations:** Surgeon to the Central-Neurological and Workhouse Hospitals, Instructor in Surgery, College of Physicians ad Surgeons, Columbia University, New York City


### ADENOMYOMA OF THE RECTUM

**By Frank C. Yeomans, M.D., F.A.C.S.,**

**Surgeon to the Central-Neurological and Workhouse Hospitals, Instructor in Surgery, College of Physicians ad Surgeons, Columbia University, New York City.**

The writer's interest in adenomyoma is due to the following case which appears to be unique:

Mrs. V. French, aged 37, the mother of two healthy children, was seen in consultation in September, 1916, for rectal hemorrhage and pain. The important points of her history were—Pulmonary tuberculosis (?) six years before, cured; menses painful, the flow diminishing; always constipated until three years ago. Then accurred attack of diarrhoea lasting five months, and thereafter intermittent attacks. Past year ten to twelve movements daily, containing blood and mucus. Pain over sacrum is aggravated at menstrual period and with the diarrhoea.

Physical Examination: Pale but well nourished woman, weighing 142 pounds. Wasserman of the blood, negative. Urine normal. Chest and abdomen negative.

Rectal Examintion: Three and one-half inches up on anterior rectal wall, just above cervix uteri, finger feels a hard, fixed, fairly tender mass, the limits of which cannot be defined Proctoscope shows a superficial ulceration, size of a quarter, at recto-sigmoidal juncture. This is red, clean, and bleeds freely on contact. Vaginal examination negative except in posterior rornix wes felt the same mass apparently the size of a guinea-hen's egg.

Operation, Sept. 26, 1917. Left rectus incisioin. No growths were found in liver or other abdominal viscera. The tumor was located in anterior wall of sigmoid just above its juncture with the rectum and extended down two inches on rectum, servix uteri and posterior vaginal wall. Lower third of sigmoid was mobilized, including small portion of posterior wall of uterus and its cervix, and superior hemorrhoidal artery was ligated. Then the abdominal wound was closed and in lithotomy position the operation was completed by a typical Genu-Tuttle extirpation of the rectum, including the posterior vaginal fornix. Patient reacted promptly from the immediate shock of the operation. Bowels acted on third day and union of sigmoid to peri-anal skin was primary except at one spot, which soon granulated. Patient left hospital in three and a half weeks and is now well, having normal anal sensibility for bowel actions, which occur once or twice daily with normal control. Vaginal and rectal examinations show no abnormalties.

Interest in this and similar tumors in this location centers in their

(a) Origin. Suggested origins are uterine mucosa, Wolffian ducts and ''embryonic rests persisting from the fusion of Miller's ducts.'' The author's case was clinically an intestinal growth. Dr. James Ewing, after careful study, reported ''the most likely origin is from superfluous material derived from that portion of the lower gut which continues on in the embryo to the bladder and allantois, and which normally atrophies. Persistence of a portion of this segment furnish a source of smooth muscle and intestinal epithelium. I do not think the tumor is of Millerian origin.''

(b) Symptoms. Varying with the development and site of the growth the symptoms would be: obstructive, dynesteric or neuralgic.

(c) Diagnosis. Tumor imparts a rather characteristic feel. Apt to be mistaken for infiltrating, inoperable malignant growth.

(d) Prognosis. Historically it is benign. Clinically it may be malignant from the symptoms to which it gives rise, or actually undergo malignant change.

(e) Treatment. Surgical removal at the earliest date possible. The abdominal route is preferable but if tumor is at recto-sigmoidal juncture a combined operation will probably be necessary for its removal, as was successfully done in the writer's case.

